# GnRH agonist-triggering ovulation in women with advanced age

**DOI:** 10.1038/s41598-022-20619-4

**Published:** 2022-09-30

**Authors:** Roni Rahav Koren, Netanella Miller, Rimon Moran, Dean Decter, Arie Berkowitz, Einat Haikin Herzberger, Amir Wiser

**Affiliations:** 1 IVF Unit, Department of Obstetrics and Gynecology, Meir Medical Center, 59 Tchernichovsky St., 4428164 Kfar Saba, Israel; 2 Sackler Faculty of Medicine, Tel Aviv University, 6997801 Tel Aviv, Israel

**Keywords:** Reproductive signs and symptoms, Reproductive disorders

## Abstract

This study evaluates the effect of GnRH agonist (GnRHa) trigger for ovulation induction among women with advanced maternal age (AMA). This is a retrospective study performed at a single assisted reproductive technology centre, 2012 to 2020. A total of 306 patients with 515 IVF cycles who were triggered with GnRHa for Ovum Pick Up (OPU), were divided into two groups according to maternal age: age ≥ 40 and age < 40. The groups were compared for demographics, stimulation parameters of IVF treatment and IVF treatment outcomes. The patients in the age < 40 group were approximately 10 years younger than the patients in the age ≥ 40 group (31 ± 5.4 vs. 41.5 ± 1.3 years, p < 0.001). The age ≥ 40 group had significantly higher mean E2/retrieved oocytes ratio, compared to the age < 40 group (310.3 ± 200.6 pg/ml vs. 239 ± 168.2 pg/ml, p = 0.003), and a lower mean MII/retrieved oocyte (35 ± 37.8 vs. 43.4 ± 35.9, p = 0.05, respectively). Multivariable logistic regression analysis for E2/retrieved oocytes demonstrated that age < 40 and total dose of gonadotropins were significant variables. In conclusion, GnRHa for ovulation triggering in high responder patients prior to OPU appears to be a good option for AMA. However, this population is characterized by different parameters of ovarian response that require further evaluation.

## Introduction

Ovarian hyperstimulation syndrome (OHSS) is a major complication of artificial reproduction technology (ART) treatments, which must be considered through careful risk assessment prior to and throughout ART treatments. Risk factors for OHSS include younger age, lower BMI, polycystic ovarian syndrome, previous episode of OHSS, increased number of follicles, increased number of retrieved oocytes, high absolute levels or rapidly rising levels of estradiol (E2), human chorionic gonadotropin (hCG) administration and pregnancy^1–5^.

In susceptible women, hCG administration for ovulation triggering and luteal support is the most important factor causing OHSS. Therefore, using GnRH agonist (GnRHa) for ovulation triggering to prevent OHSS had become routine practice in ART, making an OHSS free clinic a real concept. Furthermore, GnRHa trigger is associated with a higher rate of mature oocytes (MII oocytes), compared to hCG trigger^6–9^. However, despite the advantages of GnRHa trigger, poor reproductive outcomes have been associated with the GnRHa trigger, including lower ongoing pregnancy rate, lower live birth rate and a higher rate of miscarriage compared to hCG trigger^7,10,11^. This poor outcome is attributed to luteal phase defect and premature luteolysis because of a shorter LH surge associated with the GnRHa trigger compared to the LH surge associated with a natural cycle^12–14^.

IVF treatments among women with advanced maternal age (AMA) has increased substantially in the past years. There is limited data regarding this population^15^. It is well-established that as age increases, the number and quality of oocytes declines. This decline is pronounced at the age of ≥ 35 and is steepest at the age of ≥ 40. For this reason, age negatively correlates with the risk of developing OHSS^2,5,16^. Also, for the same reason, women with AMA are often treated with more aggressive ovarian stimulation, which may result in higher levels of serum E2 that may encourage administering a GnRHa trigger for final oocyte maturation and ovulation, instead of hCG, the gold standard trigger. Most of the studies assessing the GnRHa trigger for ovulation and oocyte maturation in order to avoid OHSS are dealing with high responder population of mainly women < 40 years. Nevertheless, a large proportion of ‘real life’ patients who come for IVF treatments do not meet the consensus of inclusion and exclusion criteria used originally in trials, and an a priori extrapolation from one population to another is done^15^. To our opinion, since data are scarce regarding women with advanced age, the same extrapolation is true when administrating GnRHa trigger to women with advanced age based on the same criteria used for OHSS prevention among younger age population. As far as we know, studies assessing the GnRHa triggering among this population do not exist.

Hence, questioning whether GnRha triggering has a similar effect among women with AMA and whether the same predictors of ovarian response used in younger age population should be used in women with AMA is imperative, for the decision to administer a GnRHa trigger.

This study aims to evaluate the results of GnRHa trigger for ovulation induction during IVF antagonist cycles, focusing on women with AMA.

## Materials and methods

### Study design

This retrospective cohort study included women at-risk for developing OHSS who were treated with a GnRH antagonist protocol and a GnRHa trigger for ovulation. Clinical and laboratory data were collected from the electronic records of IVF patients during 2012–2020. Patients 18–45 years old were included and divided into two groups according to age: 18–39 and 40–45 years. Women older than 45 years of age, women undergoing fertility preservation treatments and women undergoing IVF treatments for surrogacy were not included.

### IVF treatment protocol

All study patients were treated with GnRH antagonist protocol. The IVF antagonist protocol was started on day 2 or 3 of the menstrual cycle. Patients received daily injections of recombinant FSH (rFSH; Gonal F Merck Serono SA, Aubunne Switzerland) and/or hMG (Menopur, Ferring SA, Sainet-Prex, Switzerland). The initial gonadotropin dose was determined based on: age, BMI, antral follicle count, basal FSH and previous ovarian response. When the dominant follicle was > 13–14 mm in diameter, patients received injections of GnRH antagonist (Ganirelix MSD or Cetrorelix,, Merck). When at least 3 follicles reached ≥ 17 mm in diameter, patients were considered ready for ovum pick-up (OPU). Althogh literature is inconsistent regarding its predictive value, estradiol levels of > 3500 pg/ml are accepted as a strategy to prevent OHSS^16^. As part of maintaining an OHSS free clinic a real concept and regardless of age, our departmental policy is to administer a GnRHa for ovulation triggering, instead of a classical hCG trigger before OPU at E2 levels of 2800 pg/ml. Only patients who were triggered with GnRHa were included. All patients in our cohort were triggered with 0.2 mg GnRHa (Triptorelin; Decapeptyl, Ferring GmbH, Germany). OPU was performed 36 h later. Patients whose E_2_ level was > 4500 pg/ml on the day of GnRHa triggering or who had > 25 retrieved oocytes were scheduled for cycle segmentation by freezing all embryos and transferring on the next cycle.

### Luteal support

Due to lower clinical pregnancy rate and live birth rate associated with GnRHa trigger for ovulation in fresh ET cycles^7,10^, our departmental policy for luteal support in the case of GnRHa trigger and fresh ET, established in our previous study^17^, is to administer repeated alternate-day doses of 0.1 mg GnRHa from day 3 following OPU (total 5 doses). This luteal support is co-administered with vaginal micronized progesterone (Endometrin 100 mg, TID) from day 1 after OPU. When E2 levels are > 4500 pg/ml and/or > 25 oocytes were retrieved, a freeze-all strategy, where all embryos were frozen at blastocyst stage was performed.

### Study outcomes

The primary outcomes of this study were MII/retrieved oocytes and the clinical pregnancy rate among women ≥ 40. The secondary outcome was E2/retrieved oocytes among women < 40 years and among women ≥ 40.

### Statistical analysis

#### Sample size calculation

Epi Info software was used for sample size calculation. Based on the assumption that we accepted a 30% difference in E2/oocyte ratio among the groups, we calculated that 49 patients were required in each group to provide a power of 80%, at a 2-sided alpha level of 5%.

Data were analyzed using SPSS 24.0 for windows (SPSS, Inc., Armonk, NY, USA). Data are presented as number and percentage for nominal parameters and as mean and standard deviation for continuous variables. p-values were calculated using χ^2^ or t test and were considered significant at p < 0.05. For results that were significant or showed a statistical trend in univariate analysis, multivariable analysis was performed, using a multiple logistic regression model to assess the potential impact of those parameters on E2/retrieved oocytes.

### Ethics approval

The study was approved by the Meir Medical Center institutional Ethical Review Board (IRB), approval number MMC-0146-20 21/7/2020. All methods were carried out in accordance with the IRB guidelines and regulations. An informed consent waiver was approved by the Meir Medical Center IRB.

### Consent to participate

All authors agreed to participate in the study.

## Results

The study population consisted of 515 embryo transfers from 306 patients and included two groups. Age < 40 group refers to 427 ET from 245 patients and age ≥ 40 group refers to 88 ET from 61 patients age ≥ 40.

The sample is described in Table 1. The mean age of patients in the age < 40 group was approximately 10 years younger than the mean age of patients in the ≥ 40 group (31 ± 5.4 vs. 41.5 ± 1.3 years, p < 0.001).

**Table 1:** Description of the study sample.

Variable	Age < 40 n = 245 patients, 427 ET	Age ≥ 40 n = 61 patients, 88 ET	P-value
Age, years (mean ± SD)	31 (5.4)	41.5 (1.3)	< 0.001
Body mass index, kg/m^2^ (mean ± SD)	24.8 (5.7)	24.3 (5.3)	0.51
Gravidity (mean ± SD)	0.7 (1)	1.1 (1.9)	0.08
Parity (mean ± SD)	0.4 (0.6)	0.6 (0.9)	0.08
LH IU/L (mean ± SD)	7.6 (5.4)	5.9 (3.1)	0.001
FSH IU/L (mean ± SD)	6.8 (2.2)	7.7 (4.4)	0.08
AFC (mean ± SD)	20.1 (8.3)	11.7 (4.7)	< 0.001
Number of cycles (mean ± SD)	3.8 (2.3)	5.2 (3.4)	0.001
Primary infertility, N (%)	269 (63.4)	46 (54.1)	0.1

*AFC* antral follicle count, *FSH* follicle stimulating hormone, *LH* luteinizing hormone.

The age ≥ 40 group had lower mean antral follicular count (AFC) and a higher mean number of IVF cycles compared to the < 40 group (11.7 ± 4.7 vs. 20.1 ± 8.3, p < 0.001 and 5.2 ± 3.4 vs. 3.8 ± 2.3, respectively; p = 0.001).

Mean gravidity, parity and basal FSH demonstrated a statistical trend towards higher means among the age ≥ 40 group (Table 1). The groups had comparable BMI and infertility status (primary vs. secondary).

Table 2 demonstrates the ovarian stimulation parameters. The age ≥ 40 group received higher doses of gonadotropins (2938.9 ± 1076.8 vs. 1830.9 ± 825.9 IU, p < 0.001), had lower mean E2 levels on trigger day (3024.4 ± 1503.3 vs. 3433 ± 1356.8 pg/ml, p = 0.01), fewer follicles ≥ 15 mm on GnRHa trigger day (7.7 ± 3.6 vs. 9.7 ± 4.5, P < 0.001), fewer retrieved oocytes (12.7 ± 7.2 vs. 17.4 ± 8.6, p < 0.001), fewer MII/retrieved oocytes (35 ± 37.8 vs. 43.4 ± 35.9, p = 0.05) and higher mean E2/retrieved oocytes (310.3 ± 200.6 vs. 239 ± 168.2 pg/ml, p = 0.003). The groups were comparable regarding LH, progesterone, and endometrial thickness on trigger day (Table 2). Fertilization rate was comparable as well (51.8% among the age ≥ 40 group vs. 51.3% among age < 40 group, p = 0.8). The age ≥ 40 group had a lower rate of blastocysts than the age < 40 group (52.7% vs. 73%, respectively; p = 0.002). Rates of cycle segmentation (“freeze all”) were comparable (52.3% among the age ≥ 40 group vs. 51.8% among the age < 40 group, p = 0.9). Clinical pregnancy rate for fresh and first frozen thawed embryos of segmented cycles per number of embryo transfers were 28.3% among the age < 40 group and 10.9% among age ≥ 40 group. No OHSS cases were reported.

**Table 2:** Ovarian stimulation parameters.

Variable	Age < 40	Age ≥ 40	P-value
Total gonadotropin dose, IU (mean ± SD)	1830.9 (825.9)	2938.9 (1076.8)	< 0.001
E2 on GnRHa day, pg/ml (mean ± SD)	3433 (1356.8)	3024.4 (1503.3)	0.01
Progesterone on GnRHa day, ng/ml (mean ± SD)	0.8 (0.6)	0.9 (0.5)	0.5
LH on GnRHa day, IU/L (mean ± SD)	1.5 (1.4)	1.7 (1.3)	0.2
Follicles ≥ 15 mm on GnRHa day, (mean ± SD)	9.7 (4.5)	7.7 (3.6)	< 0.001
Retrieved oocytes (mean ± SD)	17.4 (8.6)	12.7 (7.2)	< 0.001
MII/retrieved oocytes (mean ± SD)	43.4 (35.9)	35 (37.8)	0.05
Fertilization rate (%)	51.3	51.8	0.8
E2/retrieved oocytes pg/ml (mean ± SD)	239 (168.2)	310.3 (200.6)	0.003
Endometrial thickness, mm (mean ± SD)	10.3 (2.4)	10.2 (2.2)	0.8
Freeze all, N (%)	221 (51.8)	45 (52.3)	0.9
Day 5 embryos, N (%)	317 (73)	29 (52.7)	0.002

*E2* estradiol, *GnRHa* GnRH agonist, *LH* luteinizing hormone.

Multivariable logistic regression analysis for E2/retrieved oocytes (Table 3) demonstrates that age < 40 and total dose of gonadotropins were significant variables.

**Table 3:** Multivariable logistic regression analysis for E2/retrieved oocytes.

Variable	Unstandardized B	Coefficients std. error	Standardized coefficients beta	P-value
Age < 40 years	−63.2	25.9	−0.1	0.01
Total gonadotropin dose, IU	0.02	0.009	0.1	0.002
Gravidity	2.1	10.6	0.01	0.8
Parity	−9.2	17.7	−0.03	0.6
Endometrial thickness, mm	−0.6	3.3	−0.008	0.8

## Discussion

This study reports on the effect of GnRHa ovulation triggering on stimulation and fertility parameters of women with AMA. Our findings demonstrate that women with AMA had higher levels of E2/retrieved oocytes, and lower rates of MII oocytes.

E2/oocyte ratio has been previously studied. Orvieto et al.^18^ demonstrated that a lower E2/oocyte ratio predicts higher pregnancy rate among IVF patients ages 43–45 years. Vaughan et al.^19^ showed a dramatic increase in E2/oocyte ratio for IVF patients above age 40. Aslih et al.^20^ reported E2/MII > 204 pg/ml was associated with advanced age and higher doses of gonadotropins. Preserved steroidogenesis among regularly cycling women with advanced age is well-established^21–23^. Nevertheless, the mechanisms accounting for this are less clear. Shaw et al.^24^ measured granulosa cell aromatase expression in the follicular fluid of a spontaneous cycle of older and younger women. They showed that aromatase expression was higher in older women and concluded that an up-regulation of aromatase with aging is responsible for the preservation of both serum and follicular fluid E2 levels in older women. Pacella et al.^25^ found altered follicular fluid metabolites, including higher concentrations of E2, progesterone, prostaglandin E receptor-2, cytosolic phospholipase A2, and tumor protein 53; suggesting impaired oocyte and embryo development among women with AMA.

Currently, studies assessing the E2/oocyte ratio among IVF patients include women treated with various IVF protocols; yet all were triggered with the gold standard hCG for final oocyte maturation and ovulation. Our study, as far as we know, describes a high E2/retrieved oocyte ratio following GnRHa triggering for the first time.

Maslow et al.^26^ compared maturation rates between cycles with E2 levels > 3000 pg/mL or < 3000 pg/mL of normal and low responders undergoing planned oocyte cryopreservation, treated with GnRHa triggering. The mean age of their population was 35.9 years and no difference in maturity rates was noted between the groups.

It has been shown that GnRHa triggering for final oocyte maturation and ovulation is associated with an increased rate of MII oocytes, compared to the gold standard hCG trigger^6–9^. The GnRHa trigger induces both LH and FSH surges, which resemble the natural mid-cycle gonadotropin surge and thus, is considered more physiological than the hCG trigger. Although the role of the FSH surge is not completely understood, studies attribute the higher rate of MII oocytes associated with the GnRHa trigger to the FSH surge^27–29^.

However, studies assessing the GnRHa trigger do not specifically refer to women with AMA, for whom an altered hormonal or follicular milieu, as described by Shaw et al.^24^ and Pacella et al.^25^ may further explain the lower rate of MII oocytes among women with AMA compared to women age < 40, as demonstrated in the current study. Another possible explanation for the lower maturity rate among women with AMA may be related to decreased mitochondrial gene expression and energy production. Ntostis et al.^30^ compared the transcription activity of oocytes of young women vs. those of AMA. They revealed a decrease in mitochondrial-related transcripts from GV to MII oocytes, with a much greater reduction in MII oocytes with advanced age.

Careful monitoring of the number and size of follicles with transvaginal ultrasound (TVUS), as well as serum E2 levels are essential to predict the optimal response to controlled ovarian stimulation and for OHSS prevention, since both are key factors used in the decision to withhold hCG trigger and administer GnRHa trigger instead. However, the literature is inconsistent regarding the predictive potential of each factor, in combination or alone, and validated cut-off points do not exist^16,31–37^. While E2 level > 3500 pg/ml is considered to be high-risk for OHSS^16^, E2 levels have been shown to have low clinical value in the prediction of OHSS due to a high false positive rate^33,36^. A recent review failed to demonstrate that combined monitoring with TVUS and serum E2 is more efficacious than monitoring with TVUS alone, with regard to clinical pregnancy rates and the incidence of OHSS^32^. It has been suggested that the number of retrieved oocytes is the most direct measure of ovarian response and thus, is likely to be the best predictor^4,5,33,38,39^. This is especially true for women with AMA for whom the E2/retrieved oocytes ratio is high, as was demonstrated here. Therefore, among women with AMA, E2 level as a cut-off for administrating GnRHa trigger instead of hCG trigger, is less reliable. In addition to the lower rate of MII in AMA compared to younger women, a higher E2 cut-off for GnRHa trigger may be considered.

To our knowledge, this is the first study to examine the effect of GnRHa trigger for final oocyte maturation and ovulation, on stimulation and fertility parameters focused on women with AMA. Yet, this study had some limitations. First is the lack of an hCG trigger control group. Comparing the age ≥ 40 group who were triggered with a GnRHa to similar age group who were triggered with the gold standard hCG for ovulation would help clarify whether the GnRHa trigger is associated with a relatively higher oocyte maturation rate among this age group. However, comparing the “high responders” in this age group who were therefore, treated with GnRHa for ovulation to the same age group who were treated with hCG trigger and had a lower response to ovarian stimulation, would create a selection bias. Also, comparing the two methods of triggering among the “high responders” with advanced age would pose a risk for OHSS development among those who were triggered with hCG, and thus we assumed that conducting the study in the manner that was done would provide the most reasonable and safe method to evaluate the GnRHa trigger effect among women with advanced age. In addition, this study is limited by its retrospective nature.

In conclusion, the use of GnRHa for final oocyte maturation and ovulation in AMA is efficient. However, E2 level is less reliable in predicting the ovarian response to ovarian stimulation compared to patients younger than 40 years of age, and other parameters of ovarian response (such as follicles ≥ 15 mm) should be considered in order to decide by which method to trigger ovulation. The lower rate of MII among these patients requires further study.

## Data Availability

The datasets used and/or analysed during the current study available from the corresponding author on reasonable request.

## References

[1] Practice T, Medicine R (2008). Ovarian hyperstimulation syndrome. Fertil. Steril. (Internet; American Society for Reproductive Medicine).

[2] Delvinge A, Rozenberg S (2002). Epidemiology and prevention of ovarian hyperstimulation syndrome (OHSS): A review. Hum. Reprod. Update..

[3] Navot D, Relou A, Birkenfeld A, Rabinowitz R, Brzezinski A, Margalioth EJ (1988). Risk factors and prognostic variables in the ovarian hyperstimulation syndrome. Am. J. Obstet. Gynecol..

[4] Asch RH, Li HP, Balmaceda JP, Weckstein LN, Stone SC (1991). Severe ovarian hyperstimulation syndrome in assisted reproductive technology: Definition of high risk groups. Hum. Reprod..

[5] Enskog A, Henriksson M, Unander M, Nilsson L, Brännström M (1999). Prospective study of the clinical and laboratory parameters of patients in whom ovarian hyperstimulation syndrome developed during controlled ovarian hyperstimulation for in vitro fertilization. Fertil. Steril..

[6] Pereira N, Kelly AG, Stone LD, Witzke JD, Lekovich JP, Elias RT (2017). Gonadotropin-releasing hormone agonist trigger increases the number of oocytes and embryos available for cryopreservation in cancer patients undergoing ovarian stimulation for fertility preservation. Fertil. Steril. (Internet; Elsevier Inc.).

[7] Humaidan P, Bredkjær HE, Bungum L, Bungum M, Grøndahl ML, Westergaard L (2005). GnRH agonist (buserelin) or hCG for ovulation induction in GnRH antagonist IVF/ICSI cycles: A prospective randomized study. Hum. Reprod..

[8] Reddy J, Turan V, Bedoschi G, Moy F, Oktay K (2014). Triggering final oocyte maturation with gonadotropin-releasing hormone agonist (GnRHa) versus human chorionic gonadotropin (hCG) in breast cancer patients undergoing fertility preservation: An extended experience. J. Assist. Reprod. Genet..

[9] Pahor, M., & Manini, M.C. 基因的改变NIH Public Access. *Bone* **23**, 1–7 (2008).

[10] Youssef MAFM, Van der Veen F, Al-Inany HG, Mochtar MH, Griesinger G, NagiMohesen M (2014). Gonadotropin-releasing hormone agonist versus HCG for oocyte triggering in antagonist-assisted reproductive technology. Cochrane Database Syst. Rev..

[11] Kolibianakis EM, Schultze-Mosgau A, Schroer A, van Steirteghem A, Devroey P, Diedrich K (2005). A lower ongoing pregnancy rate can be expected when GnRH agonist is used for triggering final oocyte maturation instead of HCG in patients undergoing IVF with GnRH antagonists. Hum. Reprod..

[12] Suda T, Balakier H, Powell W, Casper RF (1990). Use of gonadotropin-releasing hormone agonist to trigger follicular maturation for in vitro fertilization. J. Clin. Endocrinol. Metab..

[13] Itskovitz J, Boldes R, Levron J, Erlik Y, Kahana L, Brandes JM (1991). Induction of preovulatory luteinizing hormone surge and prevention of ovarian hyperstimulation syndrome by gonadotropin-releasing hormone agonist. Fertil. Steril. (Internet; Elsevier Masson SAS).

[14] Humaidan P, Kol S, Papanikolaou EG (2011). GnRH agonist for triggering of final oocyte maturation: Time for a change of practice?. Hum. Reprod. Update..

[15] Hershkop E, Segal L, Fainaru O, Kol S (2017). ‘Model’ versus ‘everyday’ patients: Can randomized controlled trial data really be applied to the clinic?. Reprod. Biomed. Online (Internet; Elsevier Ltd).

[16] Pfeifer S, Butts S, Dumesic D, Fossum G, Gracia C, La Barbera A (2016). Prevention and treatment of moderate and severe ovarian hyperstimulation syndrome: A guideline. Fertil. Steril..

[17] Wiser A, Klement AH, Shavit T, Berkovitz A, Koren RR, Gonen O (2019). Repeated GnRH agonist doses for luteal support: A proof of concept. Reprod. Biomed. Online (Internet; Elsevier Ltd).

[18] Orvieto R, Bar-Hava I, Yoeli R, Ashkenazi J, Rabinerson D, Bar J (2004). Results of in vitro fertilization cycles in women aged 43–45 years. Gynecol. Endocrinol..

[19] Vaughan DA, Harrity C, Sills ES, Mocanu EV (2016). Serum estradiol:oocyte ratio as a predictor of reproductive outcome: An analysis of data from >9000 IVF cycles in the Republic of Ireland. J. Assist. Reprod. Genet..

[20] Aslih N, Michaeli M, Mashenko D, Ellenbogen A, Lebovitz O, Atzmon Y (2021). More is not always better-lower estradiol to mature oocyte ratio improved IVF outcomes. Endocr. Connect..

[21] Randolph JF, Sowers M, Bondarenko IV, Harlow SD, Luborsky JL, Little RJ (2004). Change in estradiol and follicle-stimulating hormone across the early menopausal transition: Effects of ethnicity and age. J. Clin. Endocrinol. Metab..

[22] Sowers MFR, Zheng H, McConnell D, Nan B, Harlow SD, Randolph JF (2008). Estradiol rates of change in relation to the final menstrual period in a population-based cohort of women. J. Clin. Endocrinol. Metab..

[23] Sowers MFR, Eyvazzadeh AD, McConnell D, Yosef M, Jannausch ML, Zhang D (2008). Anti-mullerian hormone and inhibin B in the definition of ovarian aging and the menopause transition. J. Clin. Endocrinol. Metab..

[24] Shaw ND, Srouji SS, Welt CK, Cox KH, Fox JH, Adams JA (2015). Compensatory increase in ovarian aromatase in older regularly cycling women. J. Clin. Endocrinol. Metab..

[25] Pacella L, Zander-Fox DL, Armstrong DT, Lane M (2012). Women with reduced ovarian reserve or advanced maternal age have an altered follicular environment. Fertil. Steril. (Internet; Elsevier Inc.).

[26] Maslow BSL, Guarnaccia M, Stefanacci C, Ramirez L, Klein JU (2020). The use of GnRH-agonist trigger for the final maturation of oocytes in normal and low responders undergoing planned oocyte cryopreservation. Hum. Reprod..

[27] Engmann L, Benadiva C, Humaidan P (2016). GnRH agonist trigger for the induction of oocyte maturation in GnRH antagonist IVF cycles: A SWOT analysis. Reprod. Biomed. Online (Internet; Elsevier Ltd).

[28] Yding AC (2002). Effect of FSH and its different isoforms on maturation of oocytes from pre-ovulatory follicles. Reprod. Biomed. Online..

[29] Haas J, Bassil R, Samara N, Zilberberg E, Mehta C, Orvieto R (2020). GnRH agonist and hCG (dual trigger) versus hCG trigger for final follicular maturation: A double-blinded, randomized controlled study. Hum. Reprod..

[30] Ntostis P, Iles D, Kokkali G, Vaxevanoglou T, Kanavakis E, Pantou A (2021). The impact of maternal age on gene expression during the GV to MII transition in euploid human oocytes. Hum. Reprod..

[31] Hur YS, Park JH, Ryu EK, Yoon HJ, Yoon SH, Hur CY (2011). Effect of artificial shrinkage on clinical outcome in fresh blastocyst transfer cycles. Clin. Exp. Reprod. Med..

[32] Kwan, I., Bhattacharya, S., Woolner, A., & Woolner, A. Monitoring of stimulated cycles in assisted reproduction (IVF and ICSI). *Cochrane Database Syst. Rev*. (Internet) **4**, CD005289. http://www.ncbi.nlm.nih.gov/pubmed/33844275. Accessed 15 May 2021 (2021). 10.1002/14651858.CD005289.pub4 PMC8094870 33844275

[33] Papanikolaou EG, Pozzobon C, Kolibianakis EM, Camus M, Tournaye H, Fatemi HM (2006). Incidence and prediction of ovarian hyperstimulation syndrome in women undergoing gonadotropin-releasing hormone antagonist in vitro fertilization cycles. Fertil. Steril..

[34] Kahnberg A, Enskog A, Brännström M, Lundin K, Bergh C (2009). Prediction of ovarian hyperstimulation syndrome in women undergoing in vitro fertilization. Acta Obstet. Gynecol. Scand..

[35] D’Angelo A, Davies R, Salah E, Nix BA, Amso NN (2004). Value of the serum estradiol level for preventing ovarian hyperstimulation syndrome: A retrospective case control study. Fertil. Steril..

[36] Hendriks DJ, Klinkert ER, Bancsi LFJMM, Looman CWN, Habbema JDF, Velde ER (2004). Use of stimulated serum estradiol measurements for the prediction of hyperresponse to ovarian stimulation in in vitro fertilization (IVF). J. Assist. Reprod. Genet..

[37] Tarlatzi TB, Venetis CA, Devreker F, Englert Y, Delbaere A (2017). What is the best predictor of severe ovarian hyperstimulation syndrome in IVF ? A cohort study. J. Assist. Reprod. Genet..

[38] Steward RG, Lan L, Shah AA, Yeh JS, Price TM, Goldfarb JM (2014). Oocyte number as a predictor for ovarian hyperstimulation syndrome and live birth: An analysis of 256,381 in vitro fertilization cycles. Fertil. Steril. (Internet; Elsevier Inc).

[39] Rahav Koren R, Gonen O, Hershko Klement A, Haikin Herzberger E, Ghetler Y, Shulman A (2018). Number of oocytes retrieved as a criterion for “freeze-all” strategy versus a single “rescue” bolus of low-dose human chorionic gonadotropin following GnRH agonist for ovulation triggering: A pilot study. Gynecol. Obstet. Invest..

